# Observations on strategies used by people with dementia to manage being assessed using validated measures: A pilot qualitative video analysis

**DOI:** 10.1111/hex.13719

**Published:** 2023-02-01

**Authors:** Alison Ward, Anne M. Jensen, Anna Camilla Ottesen, Diana S. Thoft

**Affiliations:** ^1^ Faculty of Health, Education and Society University of Northampton Northampton UK; ^2^ Act2learn Health and Social and Neuropedagogic, University College Northern Denmark Aalborg Denmark; ^3^ Applied Sciences, Department of Nursing, University College Northern Denmark Aalborg Denmark; ^4^ Research Centre of Health and Applied Technology, Research and Development, University College Northern Denmark Aalborg Denmark

**Keywords:** dementia, involvement, lifelong learning, observation, validated measures, video

## Abstract

**Background:**

Analysis of video data was conducted of validated assessments with people with dementia as part of a feasibility control study comparing a lifelong learning service with other dementia services.

**Objective:**

The aim was to provide a new perspective on what occurs during the assessment process when using validated measures in research and explore which strategies people with dementia use to manage their participation.

**Design:**

Video recordings were made of pre‐ and postintervention assessments of people with dementia. An initial pilot analysis of 10 videos of the pre‐assessments was conducted.

**Setting:**

Lifelong learning services and other dementia services situated in six municipalities in Northern Denmark took part in this study, with 55 people with dementia participating.

**Results:**

The themes identified were: ‘State of mind’ and ‘Mental resources’, showing how these aspects influenced the participants' reactions and the strategies they used.

**Discussion:**

The results are discussed in relation to how individual personality traits influence the assessment process and the way a person with dementia will manage the situation.

**Conclusion:**

The assessment situation is complex and can be influenced by the strategies adopted by individuals with dementia as they try to manage the assessment process.

**Patient or Public Contribution:**

People with dementia supported the decision‐making for the choice of validated measure used within this study.

## 1 INTRODUCTION

Growing evidence shows that people with dementia can report their views and experiences in research.^1^, ^2^ However, an area that has been little researched is how people with dementia react while being assessed using validated measures or what strategies they use in this situation. Validated measures in health research enable the assessment of the quality of care, the effectiveness of interventions and supporting decision‐making in clinical care and intervention settings.^3^ Such measures also enable an understanding of the cause and effect of health conditions and interventions. They have an important role in testing hypotheses to support decision‐making in health and social care.^3^ In dementia care and research, the use of validated measures also helps to provide a perspective on the way an individual's dementia is progressing, and therefore to understand how best to support them. Such tools are also an important part of the diagnosis. In the United Kingdom, NICE^4^ recommends the use of assessments of cognition, functional ability and mental state when diagnosing dementia. More is known about the experience of receiving a diagnosis of dementia than the impact of participating in validated measures. The communication of a diagnosis of dementia requires sensitivity, indicating that the process can be stressful and overwhelming.^5^ In Xanthopoulou and McCabe's^6^ study, participants with dementia reported they found the assessment outcome and subsequent diagnosis difficult to hear, and they were scared and upset to receive the diagnosis.

Literature on using validated measures tends to report the outcomes and rationale for their validity and use. Literature reviews on using measures with people with dementia, and more widely in using patient outcome measures, have cautioned not to burden participants or cause harm.^7^, ^8^, ^9^ However, there is little discussion about what this means in practice. Ward et al.'s^10^ review on evaluating cognitive stimulation highlighted that insufficient information is given about how the assessment of people with dementia is conducted. They also noted that little is reported on how these tests are experienced and what impact there is on the subjective interpretation of the tests by people with dementia, something that has been criticized in cognitive stimulation effect research.^11^, ^12^, ^13^ However, it is important to identify measures that are acceptable for both the research community and for those diagnosed with dementia, including reducing any impact in terms of distress, confusion, anxiety or burden in participating.^8^ Heggestad et al.^14^ argue that the assessment process can be humiliating, and people with dementia may experience a loss of dignity in taking a test. This can have a negative impact on how they see themselves and can be a reminder of the progression of their dementia. Therefore, research to explore how people with dementia experience an assessment process will provide insight to support them through this process be it for diagnostic or research purposes.

This paper provides findings from observed assessments used in a research setting. The authors provide insights into this little‐researched area, and ways to support the person with dementia, researchers and clinicians undertaking assessments. This is the second paper to present findings from this research, with the first available through Thoft et al.^15^ The first article highlighted the strategies of the researcher in undertaking assessments using validated measures. The aim of this paper is to provide a new perspective on the assessment process used in research and explore how people with dementia react and identify strategies they use when being assessed with validated measures.

## 2 MATERIALS AND METHODS

This paper presents findings from a video analysis of conducting validated measures with people with dementia. This was part of a wider feasibility and pilot study that was conducted on lifelong learning services in Denmark. Lifelong learning is an education‐led programme that provides lessons to support cognitive function, decision making and activities of daily living. It is based on the premise that people living with dementia can learn, develop and grow.^16^ The project assessed an intervention group (Lifelong Learning intervention) and a control group (treatment as usual, e.g., services at day‐care centres). The study was conducted in six municipalities in Northern Denmark. Participants were tested at the outset of the study and after 5–6 months. Participants were assessed using five validated measures: Mini‐Mental State Examination (MMSE)^17^; Quality of Life in Alzheimer's Disease Scale (QoL‐AD)^18^, ^19^; General Self‐Efficacy Scale^20^; Rosenberg Self‐Esteem Scale^21^; Hawthorn Friendship Scale.^22^ A detailed method, background to the wider study and facilitator strategies are presented in Thoft et al.^15^ and Sørensen et al.^23^ This paper provides an overview of the methods in relation to the video analysis.

### 2.1 Public and patient engagement

People with dementia and staff from the lifelong learning intervention took part in a workshop to identify the most appropriate measures to use for the wider study. Their input was gained through discussions about what they felt was important to research about the intervention and informed the final choice of validated measures used. These workshops will be the focus of future analyses.

### 2.2 Video analysis

Fifty‐five participants were recruited into the main study (*n* = 30 intervention group; *n* = 25 control group). All participants undertook pre‐ and postassessments, which were recorded using one video recorder. This was positioned to capture the participants' facial features and reactions, while also capturing the table, paperwork and side/back view of the assessor. Videos were chosen because they captured both verbal and nonverbal reactions and enabled multiple reviews of actions and behaviours which may not be identified in person.^24^

The decision to conduct an initial pilot analysis was based on the pragmatics of undertaking the analysis and testing the outcomes of this approach. Video analysis is time‐consuming, requiring multiple viewings, by several researchers. To ensure that this would elicit valuable and viable data, the team first conducted this as a pilot stage, with plans to extend this analysis. This paper, therefore, presents the findings from this initial stage. A stratified sample of 10 pre‐assessment videos was analysed. This stratification included: equal distribution across the intervention and control group (*n* = 5 per group); each locality in which the service was delivered; level of dementia (high and low MMSE‐score) and diversity of gender. The pre‐assessment videos were chosen to avoid recall or familiarity with the measures as this could be a risk if including the postassessment videos. The demographic profile of the participants from the analysed videos is reported in Table 1.

**Table 1:** Demographic of participants in analysed videos.

Video no.	Gender	Type of dementia	Age	Intervention/control	Video length
7	Male	AD	74	Control	57 min 37 s
31	Male	AD	77	Control	25 min 54 s
34	Male	OTHER	73	Intervention	58 min 30 s
49	Female	OTHER	65	Intervention	31 min 31 s
55	Male	Not specified	89	Control	37 min 30 s
60	Female	AD	62	Intervention	37 min 5 s
71	Male	OTHER	54	Control	25 min 49 s
75	Male	OTHER	74	Intervention	22 min 33 s
82	Female	OTHER	68	Control	33 min 52 s
86	Female	AD	76	Intervention	25 min 45 s

The videos were analysed using an adapted version of Ridder's^25^ video analysis approach. The identified videos were watched in full by four members of the research team to develop an analysis framework, which was tested and adapted using one video, and focused on identifying participants' reactions. The resulting framework was used to code all videos. A video graph was developed for all the videos in Excel. This notes by timeframe each reaction to the assessment situation, including physical movement, facial expressions and verbal comments, alongside researcher reflections on the action. Viewing the videos in full and reviewing this graph allowed the research team to identify clips for a deeper microanalysis that explored key moments and interactions during the assessments. These were coded alongside the verbal interaction to provide a detailed account of what occurred. Thirteen clips were chosen from the 10 videos for further microanalysis (see Table 2). The final analysis stage was to draw themes from across each microanalysis and the video graph (see Thoft et al.^15^ for further details of the method, video and participant demographics).

**Table 2:** Extract from the microanalysis.

Video no. 49; (22.07–25.12): Meaningful event: Facilitation and interpretation of questions	Assessment of event ‘I feel/think…’ or ‘The student seems to…’	Reflection of event *How can you see this response, emotions, engagement, interactions*	Supporting text *Transcription of clip*
*Description of the event, who was involved and what occurred*	*Interpretation of what occurred, taking into consideration the student's perspective*	*Researcher's reflective comments on the action*	*Extracts from the transcription*
R and P are sitting across each other around a round table. P is facing the camera. There is a question paper on the table and R has a pencil which she is using to show the questions. R asks the Self‐efficacy question. P is leaning forward and has one hand on the table holding a pencil and the other is held up against her mouth. She is looking at the paper.	It feels as though the atmosphere is relaxed and they both seem at ease.		Self‐Efficacy—When I meet a problem, I am able to identify several solutions.
P leans back and adjusts her clothing and then leans forward again in the same position. There is a pause as P thinks.	It feels as if R is giving P time to think as P seems to be concentrating on how to answer the question.	This is an example of facilitation as it recognizes that a person with dementia needs time to understand and process through their answer.	
P moves her hand from her mouth to the table and talks about a previous answer and that her answer now is going against the previous answer. She points towards the paper as she talks and is looking at the paper. There is a slight joking tone in her voice and a slight smile.	It seems that P is questioning herself and her previous answer. It does not feel as though P is upset by this as she smiles and has a jovial tone to her voice. It may be that the paper is being used by P to recall her previous answer as she points to it and is focused on the paper.	There is recognition and recall by P of her previous answers. This moment shows that P is reflecting on the answers and remembers her answers. The paper acts as visual clue to support P's attention, focus and memory.	P—Yeah, but well, it contradicts the other thing, right? But well, it can be that I should… (pointing at the paper).
Evaluation *Identifying the themes emerging from the clip*	Facilitation—Some good facilitation is shown through listening and giving time for the participant to answer and giving options for the responses. There is a dilemma in the role of the facilitator in how much to support or not during the test and how much time the participant needs to answer….		

As Table 2 demonstrates, the video analysis provides a description of the action alongside the reflections and observations of this action by the researchers. Supporting evidence is also provided through transcripts of the dialogue in the videos. The results are presented as observations that were made by the researchers with supporting statements provided in the form of descriptions of the action or participant's quotations.

The research team consisted of two senior researchers with previous experience in leading dementia‐related research, and research with the lifelong learning service in Denmark (called the Aalborg Dementia School, at The Knowledge Centre for Dementia, Aalborg Municipality). One had expertise using video analysis methods. Two other researchers completed the team, having a background in nursing and expertise in qualitative research. All members of the team undertook the analysis in Denmark working in pairs to analyse each video. The researcher with video analysis expertise provided training, with review sessions at intervals during the analysis for the team to discuss the approach and how to correctly log and review the data.

### 2.3 Ethics

Participants were recruited through their service. Each service attended a meeting with the lead researcher to inform them about the aims and process of the research. Participants were informed about the project through a participant information sheet and were able to discuss this with a member of the research team. This emphasized that their participation was voluntary and was not related to their continued use of their respective services. Participants completed a consent form before participating in both pre‐ and postdata collection phases. Where required, consent was discussed and gained with support from a family or staff member, although no proxy consent was used. All participants were self‐consenting. Danish legislation requires research studies to be based on informed consent and not on ethical approval from a national or public agency.^26^ The video recordings were not allowed to be shown outside of the research team due to the requirements of confidentiality and anonymity as stated by the Danish ethical requirements. All names used in the article are pseudonyms. In keeping with good research practice, the Regional Committee on Health Research Ethics was also consulted. It was judged that no further application was needed in relation to LBK nr 1083 of 15/09/2017 definition of a Health Science Research Project and the Committee law § 14, stk. 1, jf. § 2, nr 1‐3. These reference Danish ethical laws and recommendations of the Danish Ministry of Higher Education and Science that ensure participant safety and rights under the Danish Code of Conduct for Research Integrity.^27^

## 3 RESULTS

The 13 clips varied in length from 17 s to over 3 min. This reflected the nature of the interactions, which were often short responses to questions asked during the assessment. Two core themes were identified about the way people with dementia react and the strategies they used while being assessed using validated measures. These were: ‘State of mind’ and ‘Mental resources’.

### 3.1 State of mind

State of mind was observed as both positive and negative, with a positive outlook supporting the person with dementia to find the assessment process less stressful.

### 3.2 A positive state

An individual's state of mind could impact how they experienced and responded to being assessed. State of mind was identified through emotional state/mood, emotional responses and body language.

Participants commented on their emotional state, for example, Anni said that she is normally a ‘cheerful person’. This was also apparent in the way she presented during the assessment, especially when recalling memories of her family. She smiled and laughed as she shared her thoughts. Even when she responded incorrectly, Anni smiled while responding. For example, Anni was asked to provide the address where the assessment was taking place (MMSE), she did not know, but smiled and laughed as she recalled it was near a 10‐pin bowling alley where her husband was currently playing.

Arne also commented on his mood. He asked the researcher for feedback on whether he was responding correctly during the self‐efficacy measure. The researcher commented that there was no right or wrong answer only what Arne was feeling. Arne commented that he was in a ‘good mood’ and together they reflected that if he had been in a bad mood, it could have impacted his responses:

Arne: Yes, yes, but now I'm in a good mood today. (Smiling and laughing)

Researcher: You are right, because if you are in a bad mood, I think it would look different—don't you think?

Arne: Yes, I think. (video 75)

This exchange suggests that when in a good mood, a person may respond more positively than when in a bad mood, thus having a potential effect on the test situation. The easy relationship observed between Arne and the researcher may also have had an impact on his mood, helping to ease the test situation.

### 3.3 A negative state

The mood exhibited by participants was not always positive with some showing signs of disappointment or frustration, characterizing a more negative state of mind. For example, Lone showed disappointment when she could not recall her surname. Her body language and expression changed. She leaned forward, her smile disappeared into a sigh and she looked to the side while saying: ‘Suddenly I couldn't remember it…’ (video 31).

Even though Lone succeeded in answering the question, given time to think, her tone and body language expressed, what the research team considered disappointment. It may also have been a moment of recognition of the challenges caused by her dementia.

Participants also expressed frustration. This was mostly observed in relation to the participants' loss of ability to answer questions. This was usually directed towards themselves and their dementia. For example, Hans was telling the researcher about his former language skills:

Earlier, I had five languages (showing five fingers). I was good as hell at languages and now I can, I can just speak a little Danish … And Swedish (talks in Swedish) I can't speak that anymore—I can't understand the damn prose. And that sucks when you are on a visit there. (video 34)

During this dialogue Hans was initially relaxed in his body language, resting one arm on the table and leaning his head in the other hand, while speaking in a soft tone of voice with a slight smile on his face. This changed as he talked about his declining skills. He became increasingly restless, leaning backwards and quickly forward while pointing with his finger, brows furrowed, raising his voice and firmly placing both hands on the table. This was observed as frustration towards his failing abilities and recall of the skills he used to have.

On some occasions, participants showed contradictory verbal and nonverbal expressions. This was observed in Bo who was asked to repeat the three words in the MMSE. Bo was smiling and laughing without being seemingly happy. Bo had a tense, forced, almost unnatural smile, and although he was laughing, his body language showed nervousness or discomfort, as he was tapping his finger and moving his legs, looking away and leaning back while answering: ‘That is worse! (laughing)’ (video 7). This was observed as a reaction to not being able to answer the question.

Other verbal and nonverbal signs were observed. Examples of this include looking down at the table seeming disappointed, changing tone of voice and body language showing anger and frustration, for example, making strong hand gestures and smiling ruefully to express discomfort when being confronted with difficulties due to dementia.

### 3.4 Mental resources

Participants were observed to use the mental resources of reflection, humour and bodily movement. All the participants at times were engaged and concentrating, showing different skills to help complete the assessments.

### 3.5 Reflective skills

Reflective skills were observed in many participants. When Grethe was asked a question about her marital relationship (QoL‐AD), she replied that the responding category ‘excellent’ did not fit her usual wording; ‘It's probably excellent. No, good. I have difficulties using the word excellent—good means more to me than excellent’ (video 82). Grethe was able to reflect upon personal preferences towards the meaning of the categories showing her language and interpretation skills. The researchers experienced that several participants found it unnatural to use the category excellent.

Some of the participants also talked through their reflective process. Bente recognized that an answer she gave in the self‐efficacy questionnaire about ‘When I am confronted with a problem, I can usually find several solutions‘ contradicted her earlier answers where she said could not manage difficult situations or unexpected events:

Yeah, but well, it contradicts the other things, right but it can be that I have to change … I think about different solutions that is what I am thinking about? (fidgeting with her shirt, looking down at the paper). (video 49)

She explained that she thinks about different solutions, but assesses her abilities as ‘moderately true’ and that she can come up with solutions to her problems. Other participants reflected by comparing their abilities before their dementia diagnosis and their present abilities, and by comparing their skills to those of others. The participants would use words such as ‘before’ and ‘now’, showing that their answers were considered in light of their diagnosis. This was particularly noticed during the QoL‐AD, as Anni commented: ‘Well, normally I would say it is good enough, I think so. I don't think it's bad, my memory’ (video 86). Even though the participants were confronted with their decline, they were observed to identify several solutions on how to handle a problem when asked in the self‐efficacy test and were aware of managing dementia in their everyday lives by seeking help from others, as Bente stated: ‘I can get help’ (video 49).

### 3.6 Supporting concentration

The participants took the tests seriously, and these were completed without breaks (although these were offered), and by asking questions. Their concentration was particularly noticeable by their use of physical contact with items, such as pencils or test paper. Here the items seemed to work as a physical prompt or sensory stimulus. For example, when Knud responded to the self‐efficacy question ‘I am able to do things as good as most people’ (video 71), he was observed to follow the questions with a pencil and took time to think through his answer. The test paper for all the measures, apart from the MMSE, was placed on the table for the participant to see. Some used this, reading the questions, and pointing or touching the paper as they responded. The visual cues provided by the paper and pencil were observed to support their ability to answer.

Participants were also observed to use pauses, and look to the side before answering a question, seemingly to give their response consideration and make sure they gave an accurate account of their experience. However, looking off to the side also led to a loss of focus as the participants could lose track of the question asked.

### 3.7 Shared connection

Participants often looked at the researcher for confirmation or support when answering the questions. This sense of shared connection was also evident through their use of humour, which was observed with some participants making a joke about the question or their answer. This seemed to act as a coping strategy to mask their insecurity or difficulties in undertaking the assessment. Bente was joking about her handwriting, commenting: ‘my writing is not good’ (video 49) while apologizing to the camera, leaning back and laughing. Anni used laughter when she was not able to recall what day it was during the MMSE test: ‘Thursday? Wednesday … The days have been changed over here. Now I can't remember if its Wednesday or Thursday! (laughing)’ (video 86). This seeking confirmation and the shared humour seemed to establish a form of shared connection between the participants and the researchers.

### 3.8 Nonverbal communication

Nonverbal communication in the form of facial expressions, gestures and bodily movement was observed across all the videos. Gestures were observed as a strategy to support individuals when faced with symptoms of their dementia, for example, challenges with language. During the MMSE Hans used gestures to explain which region he lived in. He drew a map of Denmark in the air, pointing towards the Northern part of Denmark. Hans was not able to verbalize his answer so used nonverbal communication instead. Also, during the MMSE in response to which floor they were on, Arne looked out the window, gesturing to show the building was built on terraced land. By doing this, he showed awareness of the building's challenging geographical layout, even though he was not able to verbally provide the correct floor level.

Participants were also observed to use movement, fidgeting and self‐touch, for example, hugging themselves, keeping hands clasped or folded, resting them on the table, leaning backwards and forward in the chair and tapping fingers against the table. It was noted that these movements were most often used at times of potential stress.

## 4 DISCUSSION

This paper sheds light on a little researched area, to understand what takes place during a formal validated assessment process in research with people with dementia. The rationale for exploring this interaction was twofold, to provide an understanding of the assessment process and people with dementia's reactions to this, and to identify ways of providing support for the individuals at a time that could be stressful.

One of the key findings related to the way personality and mood can influence a person's response, as one participant stated, being and calling oneself a cheerful person can be a way of showing one's personality and may affect the reactions towards the assessment. This individual did not seem to react negatively regardless of whether her answers were correct or not. It may be that this participant lacked insight into the progression of their dementia and how this affected her memory. Stress, hope or personality have been reported^12^ as having the potential to impact assessment scores, while people with dementia and caregivers have identified that individual traits can influence their choices during research.^8^ How these factors can affect a score requires greater investigation, especially when these assessments are used to determine care pathways and the impact of interventions.

Another key strategy was the use of touch and movement to support people with dementia, whether this was through fidgeting, hugging themselves or touching the table and/or the answer sheet. This worked to ground the individual in the moment and act as a comfort and memory aid. People with dementia have been observed^28^ to use touch to connect in the moment and that this can support the sharing of memories, while the touch of paperwork or holding a pencil can support attention and concentration in a research context.^29^ Such connections may indicate increased physical and cognitive arousal, and fidgeting has been associated with increased motor and sensory activity in the brain.^30^ While there is limited research to explain the function of fidgeting, there appear to be links to increased neural activity and arousal^30^ that may be a physiological support mechanism for people with dementia under test‐like situations. The participants in this study were observed to fidget by tapping the table, moving their legs and making varied hand gestures, using this nonverbal communication as a way to express their emotions, both positive and negative and to support their concentration.

Stress can support our decision‐making and social interactions, however, too much can negatively impact our behaviour and our cognitive function.^31^ One way to manage stress is through tactile stimulation.^31^, ^32^ Self‐touch has also been associated as a coping mechanism for managing stress, such as hugging oneself or touching a face or hands.^31^ Skovdahl et al.^32^ describe touch as a way of supporting communication, particularly nonverbally. Therefore, the provision of a pencil or paper as a tactile object for people with dementia to use, and an understanding of body language may be a way of supporting people with dementia in undertaking an assessment and helping them to answer to the best of their abilities.

Humour was observed to work as a coping strategy when responding to the validated measures and seemingly acted to smooth over worries or tensions and to mitigate where an individual was unsure of what response to give. The use of humour to manage stressful situations, as observed in this study, has also been studied in health‐professional and patient interactions.^33^, ^34^, ^35^ Laughter can also result from a release of tension as a ‘basic biological form’ (p. 4),^36^ which helps to reduce stress and help the individual to relax. Mallett and A'Herne^37^ identified that patients, in clinical settings, used humour to deflect conflict, particularly if associated with criticism. This use of humour may be expected as people with dementia use humour as a form of tension release when under stress.^36^ However, the use of humour by people with dementia is also considered a natural part of their communication,^38^ and that humour is a strategy which is used as an expression of their ‘personhood and autonomy’ (p. 341). Humour has also been shown to make it easier for mistakes to be made, to laugh about these mistakes and to relieve stress when being with other people.^29^ While much of this research has been carried out in clinical settings, the effect of humour is similar to that which was observed in this present study and eased tense situations, supported decisions and showed individual personalities. The use of humour was a coping mechanism that could be adopted to provide a more comfortable setting and ease relationships to aid the assessment.

What is starting to be evidenced is that many factors can impact how people with dementia respond to validated measures. These factors can aid their responses but also may be detrimental. Differences in personality, mood, ways of interpreting questions or response options or responding nonverbally can all influence the final assessment score. As an example of this, in Scandinavian countries there is a cultural law—the Law of Jante—that is drawn from Sandemose^39^ and in Anglo‐Saxon societies as the ‘tall poppy syndrome’.^40^ This sets out certain personality and cultural ways of being, for example, not thinking too highly of oneself, or boastful of one's successes.^39^ In an ethnographic study^41^ of Jante, it was reported that Danes were often worried about standing out. They downplayed successes and conformed to societal norms, fearing retribution for being too boastful. The use of Likert scales that ask a participant to respond positively about one's abilities, as in this present study, therefore may be affected by this Law of Jante and how a participant responds. This law was noted by the researchers to be particularly relevant to the older generation and may have resulted in ‘good’ rather than ‘excellent’ responses, as one participant exemplified. It is therefore a question about how researchers take account of this within the way they score and report their findings. Further research is needed to understand how much these factors need to be considered and how they are managed. At present, there is little evidence that these are considered, and a possible starting point would be for researchers to monitor such factors and include this within their write‐ups so that a fuller picture develops.

Another factor worth consideration is the involvement of people with dementia in determining the core domains that led to the validated measures used in this study. This was viewed as an important aspect of the study as it ensured that the measures were reflective of the needs and experiences of those who used the service. This is not often considered when deciding on validated measures for people with dementia.^42^ Evidence from patient outcome measures research finds that such inclusive practice can lead to greater health and practice benefits, and more reliable evidence associated with the experiences of those being assessed.^43^ The production of guidance to ensure a robust and open process is followed would be a valuable resource. An example from the findings of this study also highlights the need for people with dementia to be involved in the use of and design of validated measures.

The authors acknowledge that while some findings from this study may be expected, the way that validated measures are experienced by people living with dementia is not often considered in the literature. Therefore, it is not known if or how researchers or clinicians take account of mood, personality, and so forth, when conducting assessments. The authors believe that this is an aspect that could be more openly discussed as it can impact the outcomes for evidence of the impact of an intervention, but more importantly, on the care a person with dementia receives. Only with more open conversations and research can we find a way to mitigate these variables or develop more guidance on when an assessment should or should not be used. For example, the research team are taking lessons learned from this pilot forward for a new larger‐scale evaluation of the lifelong learning service across Denmark, Norway and the United Kingom where it is now being run, and this has influenced the training provided to assessors on how to undertake the assessments.

### 4.1 Limitations

The key limitation is the number of videos analysed in this pilot analysis. The ability to generalize the findings is limited, however, this study has provided novel information on a situation that is not often researched. The identification of factors that could impact how people with dementia react and respond to validated measures warrants further investigation. People with dementia were not part of evaluating the assessment process to share what or how they had experienced the situation. This may be an area for future research so that findings are not based on observation alone but also on personal experience. A further limitation was the potential for the researchers' responses and behaviour to impact participants' responses, potentially influencing how they responded. Further research or training on how to mitigate this would be a valuable consideration for the future.

## 5 CONCLUSIONS

What has emerged is the complexities of assessing people with dementia. People with dementia are using different strategies to manage their emotional responses to being assessed. These responses may hinder or help their answers and as such this opens a potential area for further research as responses to validated measurers may not provide an absolute answer. They rather need to be considered in relation to how the individual responds physically and verbally during the assessment and their cultural background. What this study provides is insight into the assessment process, highlighting that there may be more to consider when interpreting findings from validated measures and that there are approaches that can support the person with dementia to manage what can be a potentially stressful situation.

## CONFLICT OF INTEREST STATEMENT

The authors declare no conflicts of interest.

## Supporting information

Supplementary information.

## Data Availability

Anonymized data are available on request due to privacy/ethical restrictions.
